# Impact of Tumor Genomic Mutations on Thrombotic Risk in Cancer Patients

**DOI:** 10.3390/cancers12071958

**Published:** 2020-07-19

**Authors:** Orly Leiva, Jean M. Connors, Hanny Al-Samkari

**Affiliations:** 1 Department of Medicine, Brigham and Women’s Hospital, Boston, MA 02215, USA; oleiva@partners.org; 2 Harvard Medical School, Boston, MA 02215, USA; jconnors@bwh.harvard.edu; 3 Hematology Division, Brigham and Women’s Hospital, Boston, MA 02215, USA; 4 Division of Hematology Oncology, Massachusetts General Hospital, Boston, MA 02114, USA

**Keywords:** molecular subtype, cancer, venous thromboembolism, arterial thrombosis, ALK, ROS1, KRAS

## Abstract

Venous thromboembolism (VTE) is common in patients with cancer and is an important contributor to morbidity and mortality in these patients. Early thromboprophylaxis initiated only in those cancer patients at highest risk for VTE would be optimal. Risk stratification scores incorporating tumor location, laboratory values and patient characteristics have attempted to identify those patients most likely to benefit from thromboprophylaxis but even well-validated scores are not able to reliably distinguish the highest-risk patients. Recognizing that tumor genetics affect the biology and behavior of malignancies, recent studies have explored the impact of specific molecular aberrations on the rate of VTE in cancer patients. The presence of certain molecular aberrations in a variety of different cancers, including lung, colon, brain and hematologic tumors, have been associated with an increased risk of VTE and arterial thrombotic events. This review examines the findings of these studies and discusses the implications of these findings on decisions relating to thromboprophylaxis use in the clinical setting. Ultimately, the integration of tumor molecular genomic information into clinical VTE risk stratification scores in cancer patients may prove to be a major advancement in the prevention of cancer-associated thrombosis.

## 1. Introduction

Thrombotic complications, in particular venous thromboembolism (VTE), are common in patients with cancer where they are a major cause of morbidity and mortality [1]. VTE complicates the clinical course of 5–10% of all cancer patients, with the risk being the greatest during the first year following cancer diagnosis [1,2]. Additionally, cancer patients who have had VTE events have an approximately 4-fold increased risk of death than those without VTE [3].

While thromboprophylaxis would be expected to be of high utility in cancer patients given these statistics, the elevated bleeding risk in this population precludes indiscriminate thromboprophylaxis use [4]. Among those patients receiving anticoagulation, cancer patients have an increased incidence of bleeding compared to non-cancer patients, irrespective of the anticoagulant chosen [5]. Those cancer patients with metastatic disease, gastrointestinal, gynecological or genitourinary malignancies, coagulopathy, thrombocytopenia or a recent major bleeding event have been identified as having the greatest bleeding risk [5]. Primary thromboprophylaxis in cancer patients was initially studied using low molecular weight heparin (LMWH) in the general cancer patient population.

Numerous clinical cancer-associated VTE risk stratification scores have been developed to identify cancer patients with the highest risk of VTE (Table 1). Several of the more well-known scores include the Khorana risk score (the best validated), Vienna Cancer and Thrombosis (CATS), PROTECHT, CONKO and Tic-ONCO scores [6,7,8,9,10]. These risk scores incorporate patient’s histologic tumor type and primary location, prechemotherapy blood counts (hemoglobin, white blood cell and platelets), body mass index (BMI) or performance status, chemotherapy administered and soluble markers (D-dimer and P-selectin). Additionally, the Tic-ONCO score incorporates genetic risk score of germline polymorphisms in the *F5*, *F13* and *SERPINA10* genes [10]. Due to their inability to reliably discriminate between those patients at highest risk for VTE and those with intermediate risk within some cancer types [11,12], the utility of these scores in routine clinical practice is limited.

The advent of routine molecular testing of tumor samples has allowed for the characterization of tumors beyond histological type and location. Given the foundational role of tumor genetics in the behavior and prognosis of tumors, specific mutations or mutational signatures may also play a role in thrombotic risk, and indeed recent studies are beginning to elucidate this link. This review examines the findings of these studies and discusses the implications of these findings on the decisions pertaining to thromboprophylaxis in the clinical setting. Ultimately, the integration of tumor molecular genomic information into clinical VTE risk stratification scores in cancer patients may prove to be a major advancement in the management of cancer-associated thrombosis.

## 2. Historic and Current Approaches to Patient Selection for Primary Thromboprophylaxis of Cancer-Associated Thrombosis

Several randomized controlled trials have evaluated the use of low molecular weight heparin (LMWH) for primary thromboprophylaxis in patients with cancer. Semuloparin, an ultra-low-molecular weight heparin, was studied for efficacy and safety of thromboprophylaxis in 3212 unselected patients (no risk stratification score was applied to guide selection) with locally advanced or metastatic cancer [17]. After a median follow-up period of 3.5 months, VTE occurred in 1.2% of the semuloparin group and 3.4% of the placebo group with a number needed to treat (NNT) of 45.5 and clinically relevant bleeding occurring in 2.8% of patients receiving semuloparin versus 2.0% receiving placebo [17]. Other studies of LMWH in unselected patients have similarly found NNTs in the 40–50 range [17,18,19,20,21]. When cancer patients at high risk of VTE (Khorana score of three or greater) were selected, LMWH thromboprophylaxis reduced the VTE incidence (12% in LMWH group versus 21% in observation group) but resulted in a seven-fold increase risk of bleeding [21]. Therefore, routine use of LMWH for thromboprophylaxis in cancer patients was not recommended for unselected patients in practice guidelines and is not routinely used in the United States with the possible exception of pancreatic cancer patients undergoing chemotherapy [22].

The advent of direct oral anticoagulant (DOAC) therapy has provided additional options for the treatment and prevention of VTE in cancer patients [13,23] although limitations on reversibility in the case of bleeding remain an ongoing concern. Two large randomized control trials, Apixaban for the Prevention of Venous Thromboembolism in High-Risk Ambulatory Cancer Patients (AVERT) and Rivaroxaban for Thromboprophylaxis in High-Risk Ambulatory Patients with Cancer (CASSINI), investigated the role of thromboprophylaxis in cancer patients deemed to be high risk of VTE (Khorana risk score of ≥ 2) [24,25]. In the AVERT trial, patients with an active malignancy undergoing chemotherapy treatment with a Khorana score of 2 or higher were randomized to apixaban at a dose of 2.5 mg twice a day or placebo for 180 days. In the intention-to-treat analysis, the apixaban group had a reduced incidence of VTE compared to the placebo group (4.2% vs. 10.2%, respectively) at the expense of increased major bleeding (3.5% vs. 1.8%) and clinically relevant non-major bleeding (7.3% vs. 5.5%). There was no difference in overall survival between the two groups, although the trial was not powered to detect a difference. The NNT to prevent VTE (incidental or symptomatic) with apixaban was 17 and the number needed to harm (NNH) for major bleeding was 59 [25]. The CASSINI trial investigated the safety and efficacy of rivaroxaban 10 mg daily in the prevention of cancer associated VTE. In contrast to AVERT, in which patients were not screened for VTE during the trial screening period, participations in CASSINI underwent venous duplex ultrasound screening of VTE in both legs prior to enrollment (and were excluded if an occult VTE was found) and also underwent ultrasound screening every 8 weeks during the study period. Additionally, CASSINI had a higher proportion of pancreatic cancer participants than AVERT (32% vs. 13%, respectively) and AVERT had slightly more patients with Khorana scores of 4 or greater than CASSINI (8.9% vs. 6.6%). In CASSINI, the intention-to-treat analysis found no significant reduction in VTE events in the rivaroxaban group compared to placebo after 180 days and no increased risk of major bleeding [24]. However, in the on-treatment analysis, rivaroxaban did significantly reduce thrombotic events compared to placebo (2.6% vs. 6.4% with a hazard ratio of 0.40). While these trials represent an improvement over the unselected population evaluated in the prior LMWH trials, the overall NNT to prevent one VTE using a Khorana score of 2 or higher and low-dose DOAC therapy was approximately 20–25, with a NNH to cause a major bleed of approximately 75 [26]. These findings suggest that use of the Khorana risk score to guide thromboprophylaxis as was done in AVERT and CASSINI offers a clear tradeoff: prevent three VTE events for every major bleed caused by thromboprophylaxis. Thus, even with the use of the best-validated existing risk-prediction model, optimal patient selection for primary thromboprophylaxis remains challenging.

## 3. Molecular Aberrations Associated with Increased Thrombotic Risk

The discovery of targetable driver mutations and distinct molecular subtypes in most common cancers has revolutionized clinical oncology. Although decades of data and study have been devoted to targeting driver tumor genomic aberrations with mutation-specific treatments and understanding how different genotypes respond to traditional cancer treatments, our understanding of the contribution of tumor genomics to thrombotic risk is still in its early stages. Nonetheless, several studies have evaluated the contribution of certain molecular aberrations in several tumor types. Specific aberrations of interest are summarized in Table 2 and described in greater detail below.

### 3.1. Lung Cancer

Lung cancer has been historically associated with an intermediate risk of VTE compared to other malignancies, with a risk of approximately 7–13% [6,44]. A meta-analysis of randomized controlled trials of thromboprophylaxis in lung cancer showed a reduction of thrombosis with prophylactic anticoagulation (NNT of 25) at the expense of increased bleeding risk and no effect on overall survival [45]. Prior studies have demonstrated that the Khorana risk score poorly discriminates between patients with lung cancer at high versus intermediate risk of VTE [11,12]. However, recent evidence has suggested that certain molecular subtypes of lung cancer may have a considerably higher thrombotic risk. Non-small cell lung cancer (NSCLC) patients with rearrangements in the anaplastic lymphoma kinase (*ALK*) and c-ros oncogene1 (*ROS1*) genes have been shown in multiple studies to have an increased risk of VTE events.

The *ALK* rearrangement occurs in 5% of NSCLC patients [46]. Hypercoagulability including recurrent thrombosis despite adequate anticoagulation and disseminated intravascular coagulation have been described in *ALK*-rearranged NSCLC [47,48,49]. Several initial studies (ranging in size from 17 to 241 patients with the *ALK* rearrangement) suggested an increased risk of VTE in *ALK*-rearranged NSCLC, although findings were not consistent (VTE rates ranged from 8% to 47%, Table 3) [27,28,29,30,31,32,35,50]. A recently-published large cohort study (807 advanced NSCLC patients, including 422 patients with the *ALK* rearrangement and 385 without the *ALK* rearrangement as a control group) utilizing multivariable time-to-event regression analyses (Cox proportional hazards model) and multivariable time-to-event regression analyses accounting for the competing risk of death (model of Fine and Gray) confirmed an increased VTE rate, finding an approximate four-fold increase in VTE risk for patients with the ALK rearrangement relative to those without (Cox model: hazard ratio (HR) 3.70 (95% CI, 2.51-5.44, *p* < 0.001); competing-risks: subhazard ratio (SHR) 3.91 (95% CI, 2.55–5.99, *p* < 0.001)) [51]. Negative binomial modeling demonstrated higher overall VTE rates in patients with the *ALK* rearrangement, reflective of the much higher rates of single and multiple VTE recurrence in this population (incidence rate ratio 2.47 (95% CI, 1.72–3.55, *p* < 0.001)) and the odds ratio (OR) for recurrent VTE was 4.85 (95% CI 2.60 to 9.52, *p* < 0.001). Additionally, utilizing similar methodology, this study also found an approximate three-fold increase in risk of arterial thrombotic events in patients with the *ALK* rearrangement compared to those without (Cox model: HR 3.15 (95% CI, 1.18–8.37, *p =* 0.021); competing-risks: SHR 2.80 (95% CI, 1.06–7.43, *p =* 0.038)). Strikingly, although patients with the *ALK* rearrangement were nearly two decades younger on average than the non-*ALK* control group, with fewer co-morbidities and lower rates of nearly every arterial and venous thrombotic risk factor, they still had higher venous and arterial thrombotic rates, strongly suggestive of a major role for the *ALK* rearrangement in increasing thrombotic risk.

*ROS1* is an oncogene that encodes an orphan receptor tyrosine kinase that is related to *ALK*. The *ROS1* rearrangement occurs in approximately 2% of NSCLC and has also been associated with increased VTE risk that may be comparable to the risk in *ALK*-rearranged lung cancer [34,52]. Patients with *ROS1-* and *ALK*-rearranged NSCLC have similar demographics, including that a majority have never smoked (77.7% and 77.2%, respectively) [34,53,54]. Incidence of thrombotic events among a retrospective cohort study involving 95 *ROS1* and 193 *ALK* rearranged NSCLC patients was 34.7% and 22.3% respectively [34]. A multivariable logistic regression analysis comparing *ROS1* and *ALK* rearranged NSCLC showed no significant difference in the odds of thrombotic events between the two groups. However, in similar analyses, *ROS1* patients had an approximately two-fold increase in odds of thrombotic events compared to patients with *EGFR-* or *KRAS*-mutant NSCLC in the same study [34]. Additionally, a subanalysis of the Crizotinib in the Pretreated Metastatic NSCL With *MET* Amplification or *ROS1* Translocation (METROS) trial including 48 *ROS1*-rearranged patients and 26 *MET*-mutated NSCLC patients demonstrated that *ROS1*-rearranged patients had an increased incidence of VTE compared to *MET*-mutated patients (41.6% vs. 15.3%) [33].

Mutations in *KRAS* and epidermal growth factor receptor (*EGFR*) genes have been studied in the context of VTE risk, with conflicting findings. Compared to patients with *ROS1* and *ALK* rearrangements, patients with *EGFR* and *KRAS* mutations had a lower risk of thrombotic events [34]. In one study of post-operative NSCLC patients, mutations in *EGFR* were associated with increased VTE risk [37]. However, another study in Chinese NSCLC patients showed that wild type *EGFR* NSCLC had a higher VTE risk than mutated *EGFR* [36]. Mutations in the *KRAS* gene have also been associated with increased VTE risk in NSCLC patients in one small trial [38]. However other trials have failed to confirm this increased VTE risk in patients with *KRAS* mutated NSCLC [28,36].

### 3.2. Colon Cancer

As with lung cancer, colorectal cancer (CRC) is associated with an intermediate risk of VTE [6]. Thromboprophylaxis in CRC may be complicated by the anti-angiogenic therapies commonly used to treat patients with metastatic disease, which can be associated with increased bleeding and thrombotic risk [55,56]. Mutations in the *KRAS* oncogene are found in approximately thirty to fifty percent of CRC [57]. *KRAS* mutations in CRC have been associated with increased risk of VTE compared to wild type *KRAS* [39]. In one study of 172 patients with metastatic CRC (65 *KRAS* mutated and 107 *KRAS* wild type), VTE occurred in 32.3% of patients with *KRAS* mutations compared to 17.8% of patients with wild type *KRAS* (odds ratio of 2.21, 95% confidence internal 1.08–4.53) [39].

### 3.3. Myeloproliferative Neoplasms

Myeloproliferative neoplasms (MPNs) are clonal stem cell disorders and include essential thrombocythemia (ET), polycythemia vera (PV) and primary myelofibrosis (PMF), among others [58]. Mutations in the Janus kinase 2 (*JAK2*), myeloproliferative leukemia virus (*MPL*) and calreticulin (*CALR*) genes are found in the majority of patients with these three classic MPNs and all lead to hyperactivity of the JAK-STAT signaling pathway normally involved in inflammatory signaling and hematopoietic cell proliferation [59]. Thrombotic and hemorrhagic complications are commonly seen in these patients with approximately 18% of patients developing thrombotic events during a 10 year period [60,61,62]. In a study of 891 patients with ET, the presence of the *JAK2* V617F mutation, the most common mutation in MPNs, was associated with a two-fold increase in the risk of VTE and arterial thrombosis compared to patients without a *JAK* 2V617F mutation (hazard ratio of 2.04 with 95% confidence interval of 1.19–3.48) [40]. Patients with PMF and *CALR* mutations or triple-negative disease (absence of *JAK2*, *MPL* or *CALR* mutations) disease have been found to have lower rates of cardiovascular events compared to patients with *JAK2* V617F mutations with rates of 0.00%, 0.80%, 0.95% and 2.52% in patients with triple negative, *CALR*-mutant, *MPL*-mutant and *JAK2* V617F-mutant disease, respectively [63]. The risk of thrombotic events is approximately two-fold lower in *CALR*-mutated PMF compared to *JAK2* V617 despite higher platelet counts and a longer overall survival in patients with *CALR* mutated PMF [41]. Though subclonal mutations in *TET2* and *ASXL1* are associated with increased risk of leukemic transformation in certain MPN patients, these mutations have not been associated with increased thrombosis risk [64,65].

### 3.4. Primary Brain Cancer

Primary brain tumors are associated with a high risk of VTE and a particularly morbid bleeding risk, making decisions about primary thromboprophylaxis more challenging [66,67]. Mutations in isocitrate dehydrogenase 1 or 2 (*IDH1/2*) are commonly found in primary brain tumors (more than 70% in grade II and III astrocytomas and oligodendrogliomas) and are associated with better prognosis [68]. In one retrospective study involving gliomas that were tested for *IDH1/2* mutations, gliomas with wild type *IDH1/2* had a cumulative incidence of VTE of 26% compared to none with mutated *IDH1/2* [42]. Additionally, increased expression of brain tumor podoplanin has been found to be associated with increased risk of VTE in patients with primary brain cancer [69]. A combination of *IDH1/2* mutation status and podoplanin expression may be helpful in identifying those patients with primary brain cancer who are at high risk of VTE with those having wild type *IDH1* and high podoplanin expression having the highest risk and those with mutant *IDH1* tumors and absent podoplanin expression having the lowest (18.2% six month risk of VTE versus 0%, respectively) [43].

## 4. Potential Mechanisms of Increased Thrombotic Risk

Tumor mutational status may influence thrombogenesis through various potential mechanisms. The tissue factor is an important physiologic trigger of coagulation and its upregulation in certain malignancies likely contributes to the prothrombotic state of malignancy [70,71]. Mutations in *KRAS* have been associated with increased tumor tissue factor expression in CRC and lung cancer [72,73]. Mutations in *IDH1* led to hypermethylation of the F3 promoter of the tissue factor gene leading to decreased expression and may explain the decreased risk of VTE in primary brain cancer patients with mutant *IDH1* [42,74]. Inflammation is known to induce a prothrombotic state and might play a role in cancer associated VTE [75,76]. The mechanisms behind *ALK* rearrangement and increased thrombotic risk in NSCLC are unclear but some studies in lymphomas with *ALK* mutations suggest the *ALK* rearrangement results in increased STAT3 signaling and inflammation [77]. *ALK* fusion proteins activate STAT3, which participates in downstream signaling of inflammatory cytokines. *ALK* has also been shown to be important in the activation of the NLRP3 inflammasome in macrophages [78]. The mechanisms behind increased thrombotic risk in MPNs may also be related to increased JAK-STAT signaling and inflammation [60]. Other potential mechanisms for thrombosis include the upregulation of lysyl oxidase (LOX), an enzyme more commonly known to be involved in collagen cross-linking but may also increase platelet reactivity and thrombosis risk [79,80,81]. Nasser and colleagues provide a comprehensive review of the mechanisms behind hypercoagulability in cancer patients that is beyond the scope of this review [82].

## 5. Implications of Tumor Molecular Aberrations on the Use of Primary Thromboprophylaxis

Balancing the scales of bleeding and thrombotic risk in the cancer patient presents unique challenges [83]. Although we have the various cancer-associated VTE risk stratification scores, the present and future bleeding risk must be considered, including risks associated with radiation therapy, chemotherapy-induced thrombocytopenia and other concerns. Although most studies to date have been retrospective and observational, large cohorts have demonstrated significantly increased risk of thrombosis in patients with *ALK* and *ROS1* rearranged NSCLC compared to those without those rearrangements. Therefore, clinicians may contemplate a lower threshold to consider thromboprophylaxis in patients with NSCLC and either *ALK* or *ROS1* rearrangement who otherwise have traditional risk scores in the intermediate risk range. Similarly, in the case of molecular aberrations in other tumors (Table 2), knowledge that a patient may be at increased thrombotic risk due to their underlying tumor genotype is another piece of information that the treating clinician can consider when determining if a patient may be likely to benefit from thromboprophylaxis, along with traditional thrombotic risk factors such as elevated BMI, previous VTE and known hereditary thrombophilia. Further studies on incorporating tumor molecular aberrations into traditional risk scores may enhance the ability of risk scores to identify the patients most likely to benefit from primary thromboprophylaxis.

## 6. Conclusions

Thrombosis greatly contributes to morbidity and mortality in cancer patients. The use of thromboprophylaxis in clinical practice is based on the balance of benefit and risk of bleeding. Despite recent advances in primary thromboprophylaxis of cancer patients, the current tools available for patient selection are suboptimal given increased bleeding risk that may outweigh any benefit [26]. Available risk stratification scores appear to be inadequate for many groups of patients [11,12]. A reason for this could be the lack of consideration of underlying tumor biology mutational status of the primary tumor. Molecular aberrations involving various driver mutations including *ALK*, *ROS1*, *KRAS*, *IDH1/2* and *JAK2* may impact thrombotic risk in various tumor types. Additional study is needed to understand the precise role that tumor genetics plays in the risk of venous and arterial thrombotic events across the spectrum of malignancies. This could allow for the refinement of clinical risk stratification tools that could improve our ability to select patients for primary thromboprophylaxis.

## Figures and Tables

**Table 1:** Risk stratification models for venous thromboembolism (VTE) risk in cancer patients. Adapted with permission from Song et al. [13].

Score	Incorporated Risk Factors
*Khorana score (KS)* [14]	Tumor site of origin: Very high risk: stomach, pancreas High risk: lung, lymphoma, gynecologic, bladder, testicular Prechemotherapy platelet count ≥350 × 10^9^/L Hemoglobin level < 10 g/dL or use of erythropoiesis-stimulating agents Prechemotherapy leukocyte count > 11 × 10^9^/L Body mass index ≥35 kg/m^2^
*Vienna CATS score* [7]	KS plus the following: Soluble P-selectin >53.1 ng/L D-dimer ≥1.44 µg/L
*PROTECHT score* [8]	KS plus the following: Use of platinum-based therapy Use of gemcitabine
*CONKO score* [9]	Tumor site of origin: Very high risk: stomach, pancreas High risk: lung, lymphoma, gynecologic, bladder, testicular Prechemotherapy platelet count ≥350 × 10^9^/L Hemoglobin level < 10 g/dL or use of erythropoiesis-stimulating agents Prechemotherapy leukocyte count > 11 × 10^9^/L WHO performance status ≥2
*Tic-ONCO score* [10]	Tumor site of origin: Very high risk: stomach, pancreas High risk: lung, lymphoma, gynecologic, bladder, testicular Genetic risk score (germline polymorphisms in *F5*, *F13* or *SERPINA10*) Body mass index > 25 kg/m^2^ Family history of VTE
*ONKOTEV score* [15]	KS > 2 Metastatic cancer Personal history of VTE Macroscopic vascular or lymphatic compression
*COMPASS-CAT score* [16]	Breast, lung, ovarian or colorectal cancer only Cancer-related risk factors: Anthracycline or anti-hormonal therapy in women with breast cancer Time since cancer diagnosis ≤6 months Central venous catheter Advanced cancer stage Predisposing risk factors: Cardiovascular risk factors (≥2 of peripheral artery disease, ischemic stroke, coronary artery disease, hypertension, hyperlipidemia, diabetes, obesity) Recent hospitalization for acute medical illness Personal history of VTE Prechemotherapy platelet count ≥350 × 10^9^/L

**Table 2:** Molecular aberrations associated with increased thromboembolic risk.

Genetic Mutation	Tumor type	Incidence of VTE	Comments	References
*ALK* rearrangement	Lung	26.9% to 47.1%	2.2-to-5-fold increase compared to no *ALK* rearrangement	[27,28,29,30,31,32]
*ROS1* rearrangement	Lung	34.6% to 41.6%	3-to-5-fold increase compared to no *ROS1* rearrangement	[33,34]
*EGFR* mutation	Lung	9% to 35%	Data conflicts; also associated with possible reduced risk of VTE	[28,32,35,36,37,38]
*KRAS* mutation	Lung, colon	16.1% to 54%	2.6-fold increase	[28,36,38,39]
*JAK2* V617F mutation	Hematopoietic (myeloproliferative neoplasm)	12%	2-fold increase compared to *CALR* or triple negative	[40,41]
*IDH1/IDH2* wild type	Brain	18.2% to 25.6%	Mutant *IDH1/IDH2* associated with decreased VTE risk	[42,43]

**Table 3:** *ALK* Rearrangement in Non-Small Cell Lung Cancer (NSCLC) is Associated with Increased Thrombotic Risk.

Study	Study Design	N of Patients with ALK	Median Follow-Up	VTE Rate	Arterial Thrombosis Rate	Hazard Ratio vs. Non-ALK Rearranged	Comments
Zer et al. [27]	Retrospective	55	22 months	42%	Not reported		Small number of patients, did not employ survival analysis
Verso et al. [28]	Retrospective	17	Not mentioned	47.1%	Not reported	2.06 (95% CI 1.08–3.55)	Stage III and IV patients, only included pulmonary emboli. ALK rearrangement, KRAS and EGFR mutations compared to wild type. Only ALK rearrangement had significant increased risk of PE compared to wild type
Lee et al. [29]	Retrospective	24	45.6 months	Not reported	Not reported	2.45	Small number of patients, only HR reported (VTE rate not reported)
Dou et al. [30]	Prospective	26	7.5 months	26.9%	Not reported	2.47 (95% CI 1.04–5.90)	Short follow-up, only prospective study, but small number of patients with *ALK* rearrangement. Multivariable time-to-event analyses.
Zugazagoitia et al. [31]	Retrospective	241	30 months	30%	5%	Not reported	Only included patients with stage III and IV ALK rearrangements and no control group. Studied arterial events.
Ng et al. [34]	Retrospective	193	19.9 months	22.3%	9.8%	ROS1 vs ALK rearrangement: 1.45 (95% CI 0.79–2.64)	Compared *ALK* rearranged, *ROS1* rearranged, KRAS and EGFR NSCLC. Multivariable logistic regression analysis.
Gervaso et al. [32]	Retrospective	46	33.1 months	43.5%	Not reported	Not reported	Compared *ALK* rearranged, EGFR and wild type NSCLC patients. urvival analysis
Al-Samkari et al. [51]	Retrospective	422	31 months	42.7%	5%	VTE: 3.70 (95% CI, 2.51–5.44)Arterial: 3.15 (95% CI, 1.18–8.37)	Non-resectable stage III and IV *ALK* rearranged and non-*ALK*-rearranged NSCLC. Employed multivariable time-to-event analyses (Cox proportional hazards, competing risks model of Fine and Gray).
